# Identification and Characterization of Nasal Polyposis and Mycoplasma Superinfection by Scanning Electron Microscopy and Nasal Cytology with Optical Microscopy: A Case Report

**DOI:** 10.3390/diagnostics9040174

**Published:** 2019-11-04

**Authors:** Arturo Armone Caruso, Veronica Viola, Salvatore Del Prete, Sabato Leo, Daniela Marasco, Andrea Fulgione, Daniele Naviglio, Monica Gallo

**Affiliations:** 1 ENT Department, AIAS Structure of Afragola, Contrada Leutrec snc, 80021 Naples, Italy; armocar@libero.it; 2 Department of Chemical Sciences, University of Naples Federico II, via Cintia, 21, 80126 Naples, Italy; violaveronica@outlook.it; 3 Service Biotech s.r.l., 80132 Napoli, Italy; saldep@gmail.com (S.D.P.); danielamarasco.servicebiotech@gmail.com (D.M.); 4 ENT Outpatient Clinic at Scafati Hospital ASL SA, 84018 Salerno, Italy; sabato.leo@libero.it; 5 Department of Agricultural Science, University of Naples Federico II, Via Università 100, 80045 Portici (NA), Italy; andrea.fulgione@unina.it; 6 Department of Molecular Medicine and Medical Biotechnology, University of Naples Federico II, via Pansini, 5, 80131 Naples, Italy

**Keywords:** mycoplasma, nasal cytology, nasal polyposis, optical microscopy, scanning electron microscopy

## Abstract

Nasal polyposis is characterized by benign, non-cancerous and painless growths originating in the tissue of the nasal cavities and paranasal sinuses. Polyps arise from chronic inflammation due to asthma, recurrent infections, allergies, drug sensitivity or immune disorders. They can obstruct the nasal cavities and thus cause respiratory problems, a reduction in the sense of smell and susceptibility to infections. Furthermore, nasal polyps can recur. Hence the importance of using valid diagnostic methods. In this work, the diagnostic investigation carried out by scanning electron microscopy (SEM) and nasal cytology led, for the first time, to the identification of a mycoplasma superinfection on nasal polyposis.

## 1. Introduction

Nasal polyposis (NP) is a common disease in 4% of the population. The nasal polyps derive from an inflammatory process of the mucosa that covers the nasal cavity wall. The polyps tend to grow and, therefore, to obstruct the nasal passages and the paranasal sinuses. The underlying causes of polyp formation are not yet fully known. However, any condition that triggers chronic inflammation in the nasal cavities or sinuses, such as infections, allergies and cellular rhinitis, can increase the risk of polyp formation [1,2,3]. Although it is a minor illness, nasal polyposis has a very negative influence on patients’ well-being, often influencing their lifestyle [4,5,6]. Moreover, the disease, although treatable, is burdened with frequent recurrences. In this work, for the first time, a case of a patient with nasal polyposis and mycoplasma [7] superinfection has been reported, in which the clinical investigation was performed using the scanning electron microscope (SEM) and the optical microscope for nasal cytology.

## 2. Clinical Case

**Figure 1:**
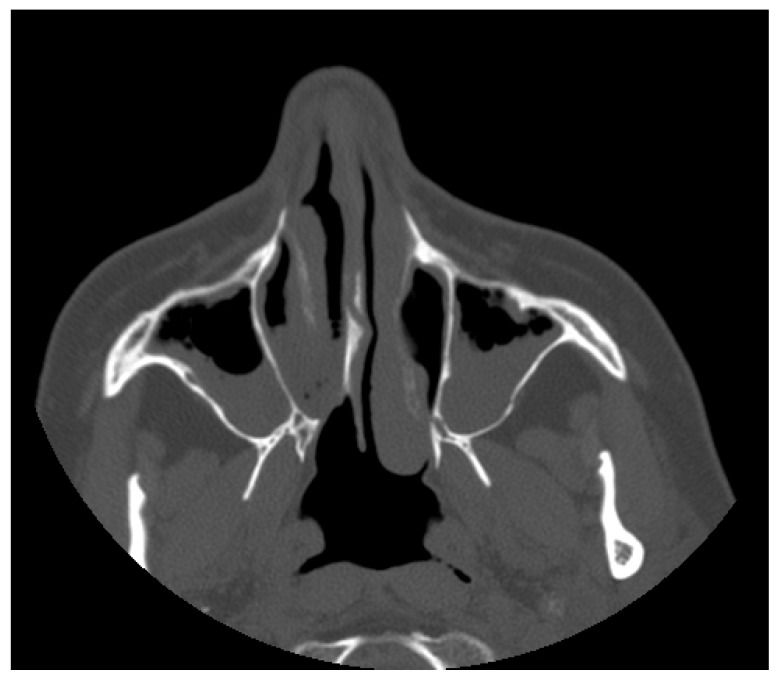
Axial scan of the facial massif where the polypoid formation is seen in both nostrils and maxillary sinuses.

A 55-year-old female was observed due to significant breathing difficulties. After the completion of the rite of privacy and informed consent to the carrying out of investigations, the physical examination was performed by means of a Nasopharyngoscope XION Medical (Berlin, Germany) diameter of 4 mm previously disinfected, according to the regulations in force. The examination documented the presence of nasal polyposis bilaterally, confirmed by axial scan (Figure 1).

Furthermore, considering some characteristics of the physical examination (presence of exudate), nasal mucosa samples were taken by nasal scraping^®^. To establish the criterion of recurrence of the polyposis itself, the sampling was applied to a polarized and colored slide according to the Pappernheim (May-Grunwald-Giemsa) panoptic, and then observed under an optical microscope at 100× magnification in oil immersion. In addition, a second sampling was performed for nasal scraping^®^ and placed on a 13 mm round slide for Scanning Electron Microscopy (SEM).

Subsequently, a nasal swab was performed and placed in Brain-Heart totipotent liquid growth culture medium. This swab was placed in 48 h culture and then scanned in electron microscopy, as per practices described in Materials and Methods.

## 3. Mycoplasma and Superantigens

Mycoplasma is a kind of bacterial pathogen that causes vertebrate diseases. Due to its size, diagnosis by light microscopy is not easy. Currently, X-rays are performed, followed by serology, and amplification of the PCR and/or culture, but all these investigations are particularly difficult at an early stage of the disease. Conversely, thanks to its deep scanning capability, SEM can observe the entire internal structure of the mycoplasma cells. A study by Sato et al. (2012) shows the applicability of the atmospheric scanning electron microscope for the study of mycoplasmas and for the diagnosis of mycoplasma infection at an early stage [8]. As is known, SEM does not exploit photons of light such as optical microscopy but uses an electron beam that strikes the sample. Thanks to the wavelength of electrons much lower than that of photons, the resolving power of a SEM is much higher than that of an optical microscope. Moreover, even the depth of field of an SEM is much higher allowing perfect images even for three-dimensional samples, with a high thickness [9,10]. Mycoplasma is “presented” to T-cells and recognized by a mechanism that is common to superantigens (bacterial, viral and neoplastic), while it is different from that of common soluble antigens. As is known, superantigens, even though they are not mitogenic sensu strictiori, induce the proliferation of T-lymphocytes even at low concentrations (lower than those of soluble antigens). Based on the results of experimental research, the mechanisms by which it is thought that mycoplasma can contribute to the development of human diseases, in genetically predisposed subjects, are essentially the following: (1) “Acute” release of cytokines; (2) Induction of autoimmunity; (3) Immuno-depression; (4) Selection of the T-lymphocyte population. These toxins are, in fact, able to activate T-lymphocytes interacting with the major histocompatibility complex (MHC-II) interaction that results in the release of pro-inflammatory cytokines. Firstly, IL-5, capable of inducing chemotaxis, migration and activation of eosinophils and delaying apoptosis, when they act as superantigens in the lateral wall of the nose in the case of chronic hyperplastic sinusitis with nasal polyposis [11,12]. Variations in the immune response to mycoplasmic antigens may be responsible for various levels of disease severity. Indeed, the superantigen would be responsible for the formation of nasal polyps [13,14].

As reported in the literature, the mycoplasms [7,15] are the smallest known bacterial forms, they are devoid of a cell wall and belong to the class of mollicutes, order of the mycoplasmatales. The mycoplasms that create disturbances to the respiratory tract are usually *Mycoplasma pneumoniae*. Pneumonia occurs only in 10–15% of cases out of 12 million, and in most cases (30–40%) involves tracheobronchitis and atypical pneumonia, or 15% of all pneumonia cases. If a mycoplasma infection is suspected, various samples should be collected per culture [9,16]. A sample is used to inoculate agar media enriched with preformed protein sources, peptones or yeast and cholesterol extracts, in the form of serum. Phenol red is added to the culture medium as a pH indicator, with thallium acetate and penicillin to inhibit the growth of other microorganisms. The sample thus inoculated in agar should be placed at 5% CO_2_ and 95% air. The second soil will be passed on a two-phase soil and then passed to agar. The growth of mycoplasma agar colonies takes on a typical fried egg shape. The diagnosis of mycoplasma infection is mainly performed on clinical grounds. Bacteria can be grown from excreted samples or pharyngeal exudate swabs, but this procedure takes several weeks to produce results. There are alternative tests carried out on blood, more precisely on cold agglutinins, but this test is not always true, because many subjects are false-negative. Mycoplasms are an extracellular pathogen. It does not invade the respiratory epithelium but adheres to the host cells by means of specialized ends P1, protein adhesin, which adheres to neuraminic acid residues and is responsible for the virulence. Once adhered to the tissue they form hydrogen peroxide to dig the underlying mucosa. Some mycoplasmic antigens act as superantigens creating an aggravation of the pathology. Thanks to biomolecular methods (pCR number of options are available for conclusive detection of the presence of mycoplasmas in escretous fluid) that are usually preferred to isolation methods on soil as we described before; it is also highlighted by more complex microscopic techniques such as confocal vision, where they appear grouped in colonies with a typical fried egg appearance, or with SEM microscopy where individual colonial constituents can also be seen and often appear to interact parasitic with other bacterial forms. Under the SEM microscope, they are clearly visible as spheroidal elements with a diameter between 0.1 and 0.2 μm, often grouped in circular or dome-shaped aggregates or even in cluster formations. Finally, mycoplasma have a slower replication capacity [9] than the classic tuberculosis, while increasing the culture time up to 15 days more or less, they mainly attack immunocompromised individuals being opportunistic and non-parasitic pathogens. Therefore, these mycoplasmas are more difficult to identify and constitute a new class of pathogens [17,18,19] SEM can be an innovative and fast means for the easy identification of these pathogens.

## 4. Bacterial Biofilms

As is known, bacteria can exist in two distinct forms, planktonic or biofilm. Biofilm existence improves bacterial survival through several mechanisms. Therefore, it is the state in which 99% of bacteria prefer to exist, forming tightly organized microcolonies enclosed in the extracellular matrix [20]. Biofilms have been implicated in multiple significant infectious processes for the practice of otolaryngology, and scanning electron microscopy (SEM) is among the most recent and most reliable modalities for biofilm detection [21]. Evidence suggests that genetic modifications of the host in innate and adaptive immunity drive biofilm susceptibility leading to a subset of patients with chronic rhinosinusitis (CRS) with recurrent and recalcitrant disease [22]. In fact, while the role of biofilms in CRS has been studied for several years, the interest of the research has now been directed towards the clarification of new methods of biofilm detection, microbial diversity and new therapeutic approaches. Recent studies on biofilm superantigens aim to clarify the immunological mechanisms of inflammation of the upper airways, in particular the type 2 response observed in nasal polyposis [23].

## 5. Materials and Methods

**Figure 2:**
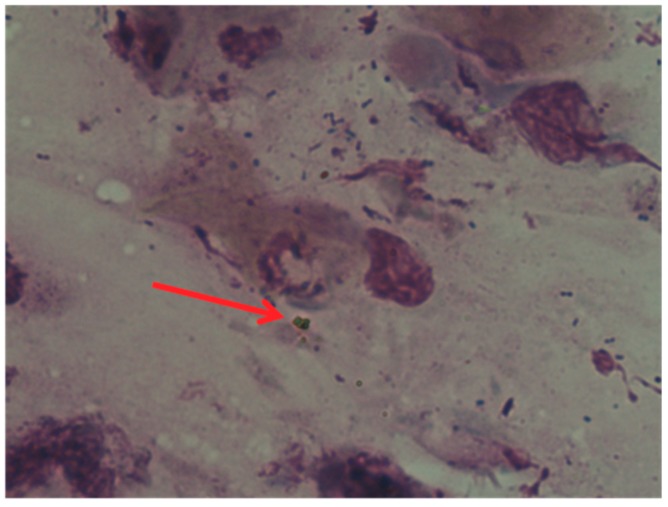
Optical microscope image. The red arrow shows the presence of masses indicating a suspected infection (100× image enlargement, oil immersion).

**Figure 3:**
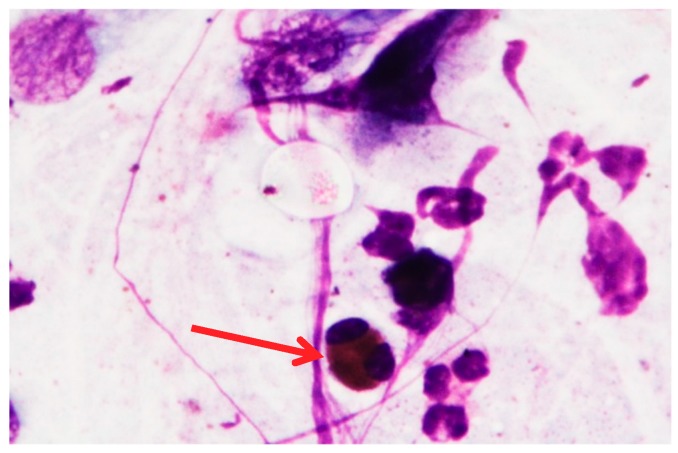
Optical microscope image. The red arrow shows the presence of eosinophils (100× image enlargement, oil immersion). In cases of allergic rhinitis, eosinophils are pathognomonic elements.

Cytological Method: a written informed consent was obtained from the patient. The cytologic sampling was carried out, after fulfilling the privacy requirements and informed consent, by means of a scraping technique, i.e., by crawling a rhino (Nasal-scraping^®^) 2–3 times on the mucous surface of the central area of the inferior turbinate. The nasal mucosal cells were placed on an electrostatically charged cytology slide (Superfrost Plus Menzel-Gläser, Thermo Scientific, Milan, Italy). The cells were then stained according to the panoptic method (3 min in pure May-Grunwald dye (Carlo Erba, Milan, Italy), 6 min in 50% May-Grunwald dye, 1 min in bidistilled water (Carlo Erba, Milan, Italy); and 30 min in Giemsa solution (Carlo Erba, Milan, Italy) diluted 1:10 *v/v*). The slide was then covered with a glass cover with dimensions of 24 × 50 mm and observed under an optical microscope (Nikon Eclipse 50i) at 100× oil-immersion enlargement. The images were recorded using a Nikon DS1 camera and digitized using a NIS-D elements computer support. Observation by optical microscope allowed to observe the presence of bacterial colonies and eosinophilic cells, using May-Grunwald-Giemsa differential staining which makes it possible to distinguish red blood cells and leukocytes, thus allowing to identify some pathologies of the blood cells (Figure 2 and Figure 3).

SEM method applied to the practice of nasal scraping^®^ cytology and buffer was grown in Brain-Heart liquid culture medium 48 h. The samples were prepared in this sense, both the nasal cytological sampling using nasal scraping^®^ and the culture, were applied on a 13 mm DIA round glass, were fixed in 2% glutaraldehyde, washed in PBS (Phosphate Buffered Saline) at pH 7.4 for 15 min for 3 times, and treated in osmio 4% for 2 h. Subsequently, the samples were subjected to 2 washings in PBS at pH 7.4 each at 30 min. Finally, the sample thus treated was dehydrated in alcohol at increasing concentrations; 30% at 25 min, 50% at 25 min, 70% at 20 min and 96% at 20 min for 2 times. Once dehydrated, the sample was placed in a critical point in CO_2_ (critical point at 31 °C and 73 atmosphere).

**Figure 4:**
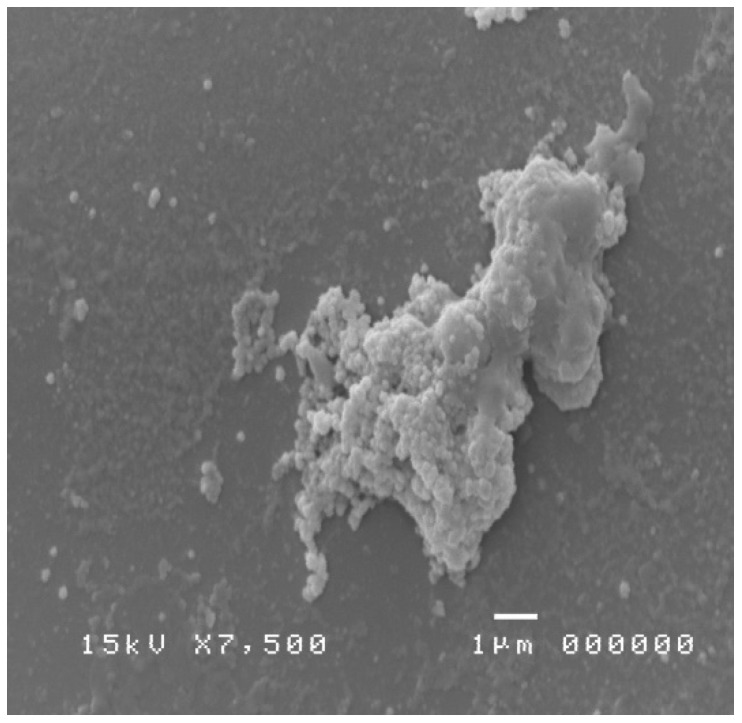
SEM image showing a mycoplasma colony (image enlargement 10,000×).

**Figure 5:**
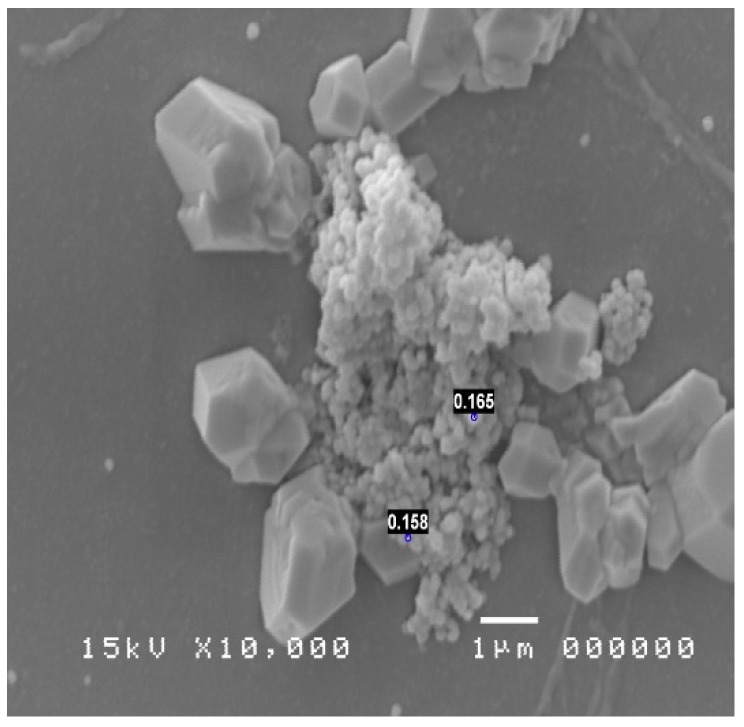
SEM image showing a colony of mycoplasma in colony caught in crystals (image enlargement 10,000×).

The treatment of the samples allowed to visualize at various magnifications the bacterial species colonizing the nasal mucosa and the typical inflammatory cells; moreover, the culture has placed attention on the pathogens involved in the phlogosis [24,25] of the nasal mucosa of the patient under examination (Figure 4 and Figure 5). These pathogens were compatible with the bacterial genus of Mycoplasmas.

## 6. Discussion

The use of a suction device to remove large volumes of mucus helps to increase the chances of obtaining representative sinus microflora. Spurious recovery of mycoplasmas, due to endoscope contamination, is possible, as well as the possibility that glutaraldehyde may not adequately kill mycoplasma. Indeed, it is known that activated alkaline glutaraldehyde is one of the most widely used high-level disinfectants worldwide. However, several reports have highlighted the potential for nontuberculous mycobacteria to develop high-level resistance to this product [26]. In our case, on the other hand, a sample was taken with a sterile swab, which demonstrated the presence of numerous small masses compatible with an infection by optical microscopy. The SEM observation has documented the presence of a spheroidal shape with dimensions between 0.176 and 0.182 microns. The forms were compatible with those of Mycoplasma. Therefore, cytochemical investigations based on the use of SEM have confirmed that the isolated bacterium belongs to this pathogen.

In conclusion, it has been observed that the patient affected by mycoplasma showed nasal polyposis [24,25,27], and presence of eosinophilic cells was justified by the role played by the ability of mycoplasma to act as superantigen [16,27]. Eosinophilic cells exist everywhere in nasal mucosa when the patient has allergic rhinitis. Therefore, in cases of allergic rhinitis, eosinophils are pathognomonic elements. Consequently, the investigations conducted showed that a comparative study of nasal cytology using optical microscopy and SEM is a precision diagnostic path that can direct the specialist towards an adequate therapy. Therefore, the use of SEM microscopy and nasal cytology represent a valid and innovative support to research and can be proposed as methods of choice in clinical diagnosis.
